# Chelating Tendencies of Bioactive Aminophosphonates

**DOI:** 10.1155/MBD.1994.247

**Published:** 1994

**Authors:** Tamas Kiss, István Lázár, Pawel Kafarski

**Affiliations:** 1 Department of Inorganic and Analytical Chemistry Kossuth Lajos University P.O. Box 21 Debrecen H -4010 Hungary; 2 Institute of Organic and Physical Chemistry Technical University Wroclaw PL 50 370 Poland

## Abstract

The metal-binding abilities of a wide variety of bioactive aminophosphonates, from the simple aminoethanephosphonic acids to the rather large macrocyclic polyaza derivatives, are discussed with special emphasis on a comparison of the analogous carboxylic acid and phosphonic  acid  systems.  Examples are  given  of  the biological importance of metal ion – aminophosphonate interactions in living systems, and also of their actual and potential applicability in medicine.


### CHELATING TENDENCIES OF BIOACTIVE AMINOPHOSPHONATES

Tamas Kiss*[1], István Lázár[1] and Pawel Kafarski[2]

[1] Department of Inorganic and Analytical Chemistry, Kossuth Lajos University, P.O. Box 21, H - 4010 Debrecen, Hungary
[2] Institute of Organic and Physical Chemistry, Technical University, PL - 50 370 Wroclaw, Poland

### Abstract

The metal-binding abilities of a wide variety of bioactive aminophosphonates, from the simple aminoethanephosphonic acids to the rather large macrocyclic polyaza derivatives, are discussed with special emphasis on a comparison of the analogous carboxylic acid and phosphonic acid systems. Examples are given of the biological importance of metal ion – aminophosphonate interactions in living systems, and also of their actual and potential applicability in medicine.

Aminophosphonic acids are broadly defined as analogues of aminocarboxylates (including polyamino and/or polycarboxylates)in which one or more carboxylic group(s) are replaced by phosphonic or related functions (phosphonous, phosphinic, etc.). A common characteristic of these compounds, in contrast to the organic phosphates, is that they all contain a carbon-to-phosphorus bond. They were first synthetized in the forties and were found to occur in living organisms in the late fifties. Only a few of them [e.g. 2-aminoethanephosphonic acid (β-AlaP), or 2-amino-3-phosphonopropionic acid (Asp-βP)] are found as free compounds in tissues, whereas they usually occur in bound forms as phosphonopeptides, phosphonolipids and phosphonoglycolipids.

The discovery of aminophosphonates in living systems stimulated the interest in this group of compounds and the intensive research directed towards synthesis of phosphonic acid analogues of protein and non-protein amino acids resulted in a new class of drugs and other bioactive compounds with a great variety of commercial applications ranging from agriculture to medicine. Consequently, studies on aminophosphonates accelerated significantly in the past twenty years, when it was revealed that many of the synthetic aminophosphonic acids display diverse and useful biological properties. Thus, this class of compounds include promising anticancer agents, strong neuromodulators, plant growth regulators and herbicides, antibacterial phosphono peptides, metal-sequestering drugs, radiopharmaceuticals and NMR imaging agents.[1-7]

What are therefore the properties of this ligand class which make them able to exert such versatile biological activity? Firstly, being structural analogues of amino acids, aminophosphonic acids can act as their antimetabolites and compete with their carboxylic counterparts for the active sites of enzymes (including metalloenzymes) and other cellular receptors. As inhibitors of metabolic processes they can exert the above-mentioned physiological activity. Secondly, aminopolyphosphonates and polyaminopolyphosphonates, including open-chain and cyclic derivatives, containing strong metal binding donors are able to chelate essential or toxic metal ions strongly enough either to compete with metalloenzymes or other metalloreceptors, or to remove toxic metal ions from living systems, or to ensure their uptake by the organism e.g. for diagnostic, or therapeutic purposes. In any cases their interactions with metal ions may be of fundamental. These features comprise the scope of this paper with special emphasis on a comparison of the chelating properties of the analogous carboxylic and phosphonic compounds.

Carboxylic and phosphonic groups differ substantially in many respects, including size (the phosphonic function is considerably larger), shape (flat carboxylate and tetrahedral phosphonate), basicity (phosphonate is more basic than carboxylate) and charge (phosphonate is binegative, while carboxylate is mononegative).

| | | |
|---|---|---|
| R<br>\|<br>CH-$NH_2$ (8.5-9.5)<br>\|<br>COOH (2.0-3.0) | R<br>\|<br>CH-$NH_2$ (9.5-10.5)<br>\|<br>$PO(OH)_2$ (5.0-6.0)<br>(0.5-1.5) | R<br>\|<br>CH-$NH_2$ (7.5-8.5)<br>\|<br>HPO(OH) (0.5-1.5) |
| α-amino-carboxylic acid | α-amino-phosphonic acid | α-amino-phosphonous acid |
| R<br>\|<br>CH-$NH_2$ (8.0-9.0)<br>\|<br>R'PO(OH) (0.5-1.5) | R<br>\|<br>CH-$NH_2$ (10.0-11.0)<br>\|<br>$CH_2$<br>\|<br>$PO(OH)_2$ (5.5-6.5)<br>(0.5-1.5) | R<br>\|<br>CH-$NH_2$ (9.5-10.5)<br>\|<br>$CH_2$<br>\|<br>$OPO(OH)_2$ (5.0-6.0)<br>(0.5-1.5) |
| α-amino-phosphinic acid | β-amino-phosphonic acid | β-amino-phosphoric acid |

**Figure 1.** Structural formulas of aminocarboxylates and their phosphono derivatives (the pK range of the donor groups are given in parenthesis).

A comparison of the acid – base characters of simple α-aminocarboxylates and their various phosphoric derivatives (see Figure 1) demonstrates that the pK of the acid-OH increases in the sequence phosphonous [–PO(H)(OH)] ~ phosphinic [–PO(R)(OH)] < carboxylic [–CO(OH)] < phosphonic [$-PO_2(OH)^-$] ~ phosphoric [$-OPO_2(OH)^-$]. The first proton of the phosphonic and phosphoric acid derivatives is very acidic and, similarly as in the phosphonous and phosphinic acids, pK ~ 1. The acidity sequence of the ammonium group of these ligands corresponds to the variations in the electron-withdrawing effects of the other group; this gives the following trend: $PO_3^{2-} < OPO_3^{2-} < CO_2^- < PO_2R^- < PO_2H^-$. Similarly as for the α-amino-carboxylate analogues, the presence of a cyclic conformation involving internal hydrogen-bonding between the phosphonate and ammonium groups in the zwitterionic form is strongly supported by the results of NMR studies.[8]

As concerns the metal-binding abilities of these phosphono derivatives, bidentate (N,O)-chelation is of greatest importance for most of the biologically important transition metal ions. Because of the relatively high basicity of the $PO_3^{2-}$ group, there is also a possibility for modentate coordination of the phosphonate group in acidic solution. In alkaline solution, monodentate $NH_2$-coordination too can occur, e.g. with cis-$Pt(NH_3)_2^{2+}$ [9-10] The sequence of stability of the (N,O)-chelated complexes corresponds roughly to the overall basicity sequence of the coordinating donor groups; the higher the basicity of the donor groups the higher the stability of the complexes formed. To illustrate this, the metal complex formation constants of the different phosphono derivatives of alanine (Ala) are compiled in Table I.

Table I. Overall stability constants and basicity adjusted stability constants[a] of the metal complexes of some aminocarboxylic acids and their phosphonic and phosphinic derivatives at I = 0.20 mol dm$^{-3}$ (KCl) and t = 25 °C

| Metal | Ligand | $\log K_{MA}$ | $\log K_{MA2}$ | $\log K_{MA}^{*}$ | $\log K_{MA2}^{*}$ |
|---|---|---|---|---|---|
| Cu(II) | α-Ala | 8.04 | 6.69 | -3.99 | -5.34 |
| | α-AlaP | 8.29 | 6.65 | -7.37 | -9.01 |
| | α-AlaPo | 4.87 | 4.04 | -4.16 | -4.99 |
| | α-AlaPi | 5.45 | 4.54 | -3.84 | -4.75 |
| Ni(II) | α-Ala | 5.32 | 4.42 | -6.71 | -7.61 |
| | α-AlaP | 5.42 | 3.89 | -10.24 | -11.77 |
| Co(II) | α-Ala | 4.24 | 3.41 | -7.79 | -8.62 |
| | α-AlaP | 4.55 | 3.15 | -11.11 | -12.51 |
| Zn(II) | α-Ala | 4.56 | 3.95 | -7.67 | -7.06 |
| | α-AlaP | 5.99 | - | -9.67 | - |
| Cu(II) | β-Ala | 6.91 | 5.45 | -6.65 | -8.11 |
| | β-AlaP | 8.53 | 6.43 | -8.60 | -10.70 |
| Co(II) | β-Ala | 3.49 | 2.59 | -10.07 | -10.97 |
| | β-AlaP | 5.16 | 3.66 | -11.97 | -13.47 |
| Ni(II) | β-Ala | 4.52 | 3.32 | -9.04 | -10.24 |
| | β-AlaP | 5.34 | 3.70 | -11.79 | -13.42 |
| Zn(II) | β-Ala | 3.78 | 3.12 | -9.78 | -10.44 |
| | β-AlaP | 6.09 | 4.85 | -11.04 | -12.28 |

[a] Values are obtained from Ref. 43, 44; $\log K_{MA}^{*} = \log K_{MA} - \Sigma pK$, characteristic for the proton displacement reaction $M^{2+} + H_nA = MA + nH^+$; $\log K_{MA_2}^{*} = \log K_{MA_2} - \Sigma pK$, characteristic for the reaction $MA + H_nA = MA_2 + nH^+$.

The equilibrium constants characteristic of the reaction $M^{2+} + H_2A = MA + 2H^+$, which express the competition between proton and metal ion for the ligand chelating site, may be a more realistic measure of the relative metal ion-binding strength. These basicity-adjusted stability constants for the aminophosphinate complexes (AlaPo and AlaPi) are about the same as those of the Ala complexes, but much larger than those of the AlaP complexes. That is, the lower overall stability of the phosphonous and phosphinic complexes is due purely to the lower basicity of the donor groups (cf. $pK_{PO_2(OH)^-}$ for the aminophosphonic acid and $pK_{PO(R)(OH)^-}$ for the aminophosphonous and aminophosphinic acids, which are 5.5 and ~1.0, respectively). However, for aminophosphonates the electrostatic and steric hindrance due to the binegative charge and the larger size of the $PO_3^{2-}$ group significantly overcompensates the stability increase arising from the higher basicity of the phosphonate group, and the proton displacement constants are 2-3 orders of magnitude lower than those for the metal complexes of the aminocarboxylate analogue, Ala.[11] It can also be seen from these basicityadjusted stability constants that the metal-binding abilities of aminophosphonates and aminocarboxylates forming five- or six-membered chelate rings follow the sequence $5(NH_2,CO_2^-) > 6(NH_2,CO_2^-) \sim 5(NH_2,PO_3^{2-}) > 6(NH_2,PO_3^{2-})$.

The presence of further coordinating donors in the molecules may modify the metal-binding abilities of these ligands. When an acetate function is attached to a simple α-aminophosphonate (GlyP) via the N-donor, forming an imino (N-phosphonomethyl-glycine, Ima-mP) or nitrilo (N,N-diphosphonomethylglycine, Nma-dP) derivative, the ligands become much stronger metal ion binders due to their tridentate or tetradentate coordination through the formation of joint chelate systems.[12]

(a)

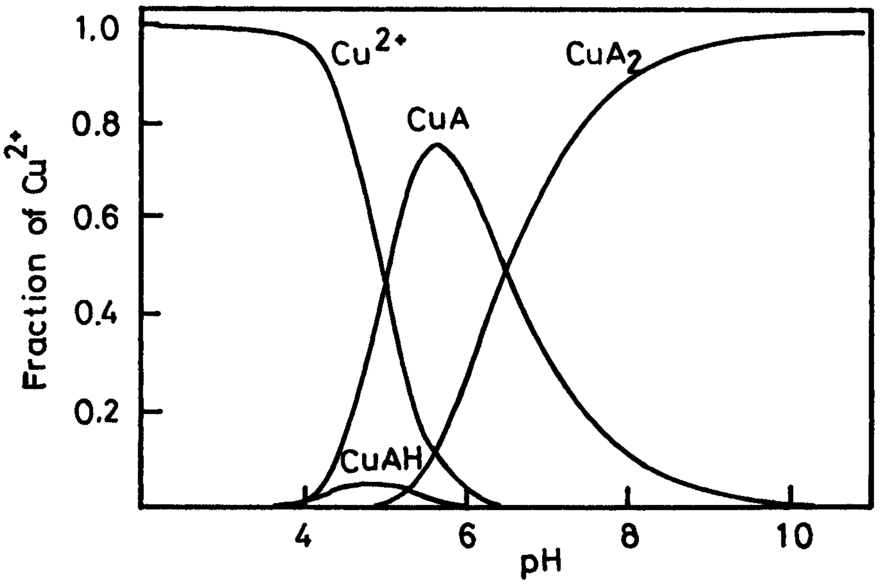

(b)

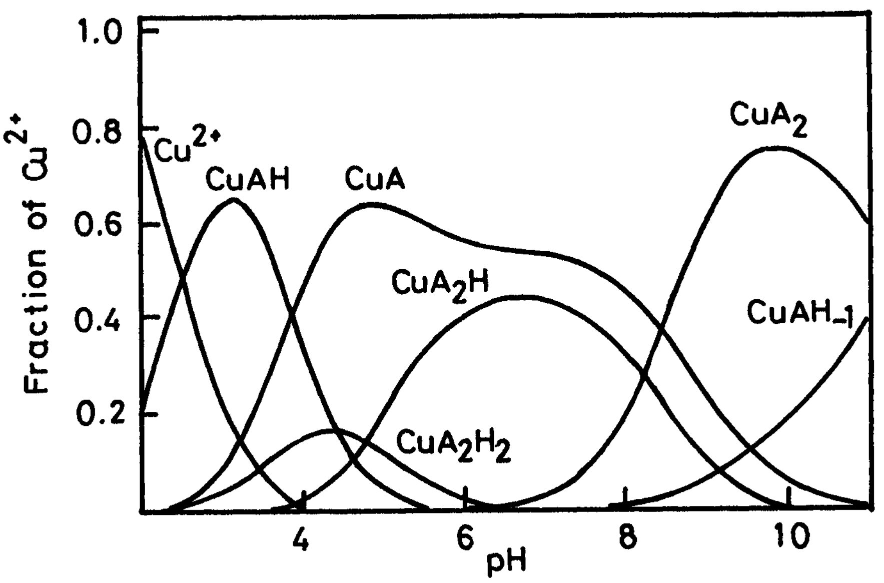

(c)

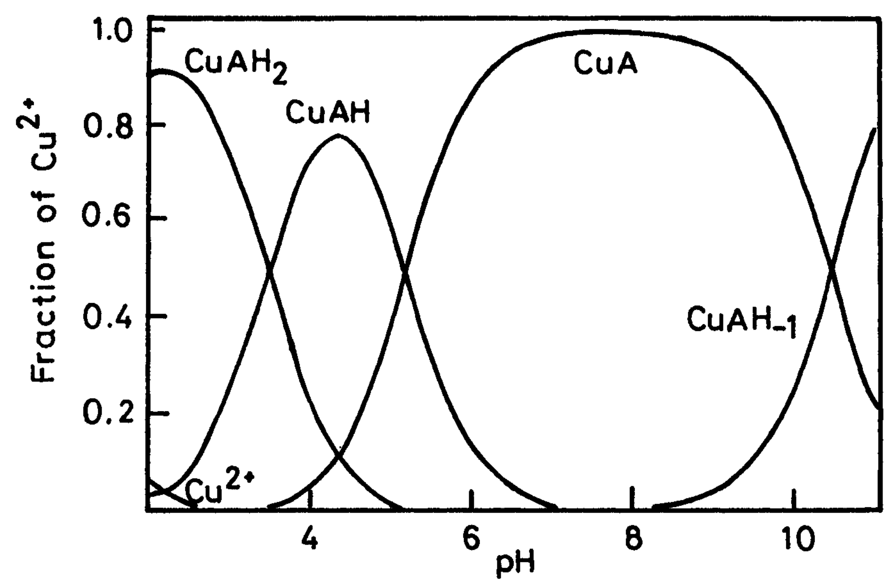

**Figure 2.** Concentration distribution curves for the complexes formed in the (a) copper(II) – 2-aminoetanephosphonic acid (AlaP), (b) copper(II) – N-(phosphono-methyl)glycine (Ima-mP), and (c) copper(II) – N-di(phosphonomethyl)-glycine (Nma-dP) systems $C_{Cu}$ = 0.002 mol dm$^{-3}$, $C_{ligand}$ = 0.004 mol dm$^{-3}$.

This is demonstrated by the speciation diagrams of these copper(II)-ligand systems depicted in Figure 2, and also reflected in the pM values (negative logarithm of the free metal ion concentration) calculated for physiological pH at mM concentration and at a metal ion to ligand ratio of 1:5, which are as follows: 8.1 for Cu(II) – AlaP, 10.2 for Cu(II) – Ima-mP, 13.5 for Cu(II) – Nma-dP, 10.5 for Cu(II) – Ida and 11.6 for Cu(II) – Nta. N-phosphonomethylglycine or glyphosate is a popular preemergence herbicide. Besides of its direct action on enolpyruvylshikimate-3-phosphate synthase another postulated mechanism of its action may be the complexation of essential metal ions in plant tissues. It should be mentioned that these mixed carboxylate-phosphonate derivatives are generally less effective metal binders than their pure carboxylate counterparts in the acidic pH range (see Figure 2) for the above mentioned reasons: the larger space requirement and higher charge of the $PO_3^{2-}$ group(s). However, there is no significant difference in their metal binding ability in the physiological pH range (cf. pM values of the respective analogues).

When an extra carboxylate function is attached to the α-aminophosphonate molecule via the carbon chain, as in the phosphonic derivatives of aspartic acid (Asp) and glutamic acid (Glu), the ligands display typical ambidentate (aminocarboxylate and aminophosphonate) coordinating capability towards transition metal ions. The coordination mode of the ligands will be governed primarily by the relative stability sequence of the ($NH_2$,$PO_3^{2-}$) and ($NH_2$,$CO_2^-$) chelates of different ring size (see above). For instance, for the two possible phosphono analogues of Asp (obtained either by substitution of its α- or β-carboxylic moiety by a phosphonic group), it was found that aminocarboxylate and aminophosphonate coordination can occur simultaneously. This results in the coexistence of different binding isomers in solution (see Figure 3). This behaviour was more marked for the analogue in which the α-carboxylate moiety was replaced by a phosphonate group.[13] This is in good accordance with the above mentioned stability trend *versus* bonding mode and chelate ring size of the complexes.

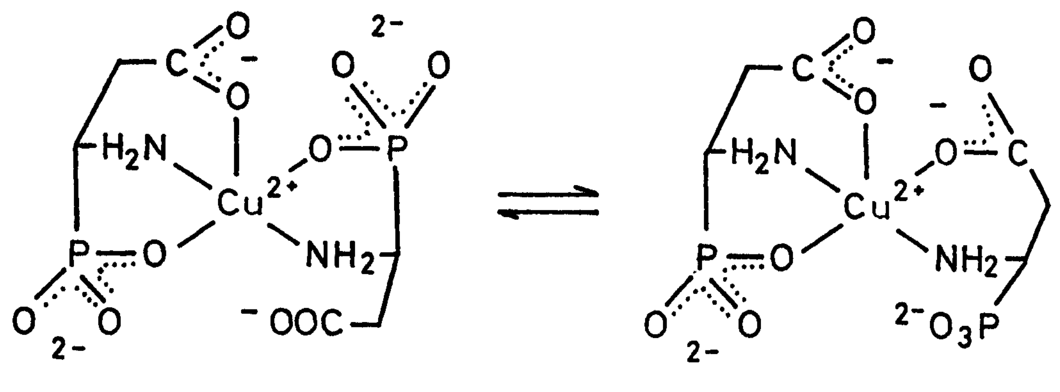

**Figure 3.** Binding isomers of the bis complex $CuA_2$ formed in the copper(II) – Asp-$\alpha$P system.

As mentioned earlier, aminophosphonic acids are potent inhibitors of many enzymes, mostly those involved in metabolism of amino acids. A good example is the interaction of the phosphonic analogues of 3,4-dihydroxyphenylalanine (Dopa) with tyrosinase, an enzyme containing four copper ions bound in the active site. A comparative study of the complex-forming properties of Dopa, DopaP [1-amino-1-(3',4'-dihydroxyphenyl)ethylphosphonic acid], and the phosphonic derivatives of 3,4-dihydroxyphenylglycine [1-amino-1-(3',4'-dihydroxyphenyl)methylphosphonic acid, DopgP] (see Figure 4) with copper(II) revealed a significant similarity in the metal-binding abilities of these ligands.

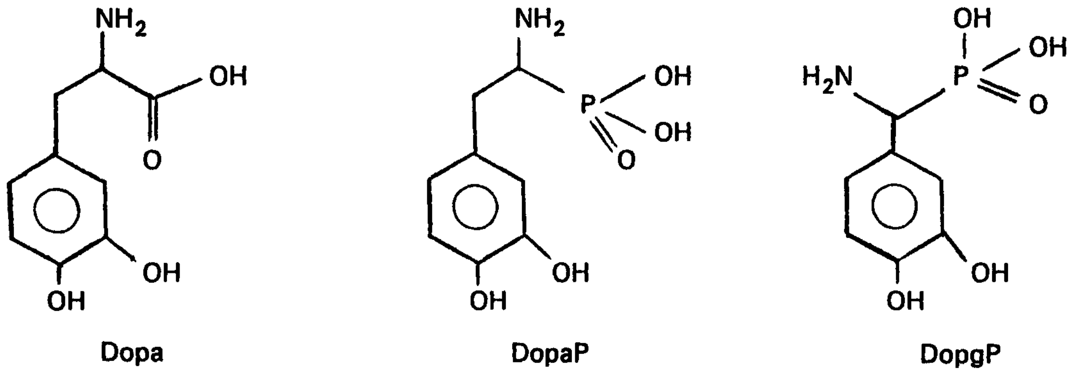

**Figure 4.** Structural formulas of some catechol derivatives.

As can be seen in Figure 4, all these ligands contain two separated chelating functions: the aminocarboxylate/aminophosphonate side-chain donors and the catecholate-O,O donors on the aromatic moiety. Speciation diagrams of Dopa and DopaP with copper(II) (see Figure 5), demonstrate that these ligands coordinate via the side-chain donors at lower pH, but via the catecholate function at higher pH. Of course, there is a possibility of the simultaneous ligand coordination via both binding sites in the case of a ligand excess and of simultaneous metal ion coordination to both binding sites in the case of a metal excess. Such type of monomeric and oligomeric species involving mixed bonding mode are formed in the intermediate pH range. The only significant difference due to the $PO_3^{2-}/CO_2^-$ substitution is that the formation of the oligomeric species of mixed bonding mode is less favoured with the aminophosphonate analogue (it can be detected by EPR only in equimolar solution).Thus, it is not surprising that DopaP serves as a synthetic substrate for tyrosinase and it does not differentiate between the carboxylate and phosphonic functions. A marked change in activity was achieved, by shortening the side chain by one methylene group, and the phosphonic derivative of Dopg proved to be one of the most powerful known inhibitors of this enzyme. However, DopgP does not display a very different metal-binding ability towards copper(II) (see Figure 5), although the EPR parameters suggest tetramer formation for both phosphonic derivative instead of a dimer, what was proposed for Dopa.

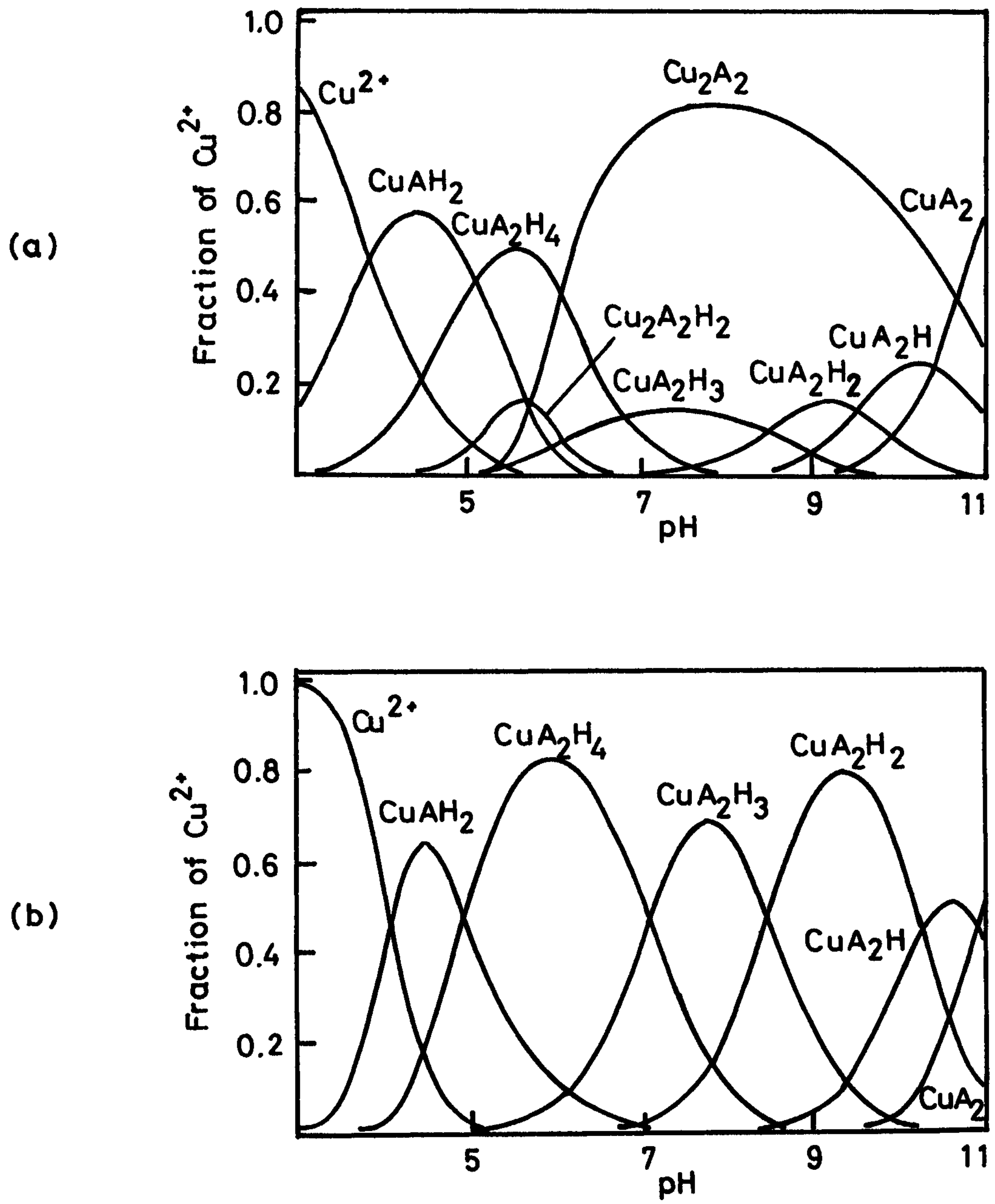

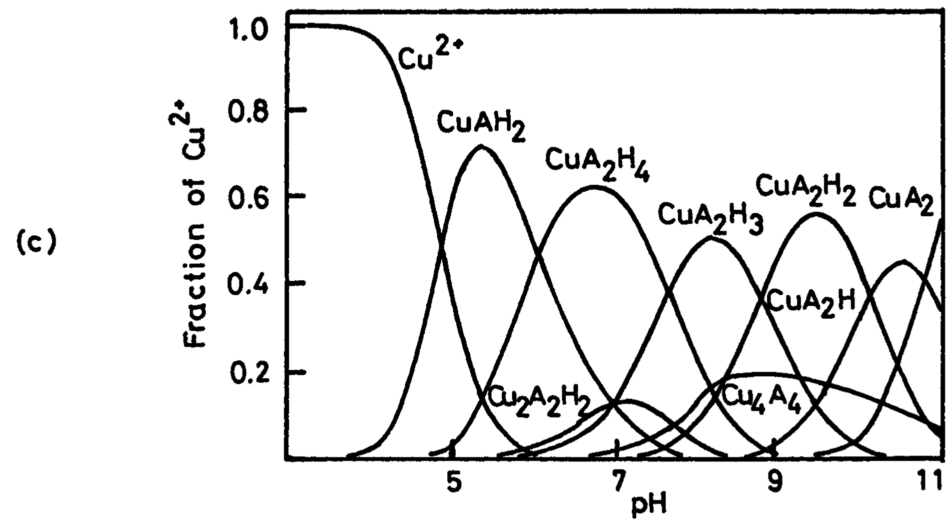

**Figure 5.** Concentration distribution curves for the complexes formed in the (a) copper(II) – L-Dopa, (b) copper(II) – L-DopaP, and (c) copper(II) – DopgP systems, $C_{Cu}$ = 0.002 mol $dm^{-3}$, $C_{ligand}$ = 0.004 mol $dm^{-3}$.

In the cyclic dimer, the magnetic coupling between the copper(II) centres is strong enough to cause a seven-line splitting of the EPR signal, while in a cyclic tetramer, as found in the copper(II)-adrenaline system, the weaker copper(II)-copper(II) interaction results in a broad, poorly-resolved signal (see Figure 6).

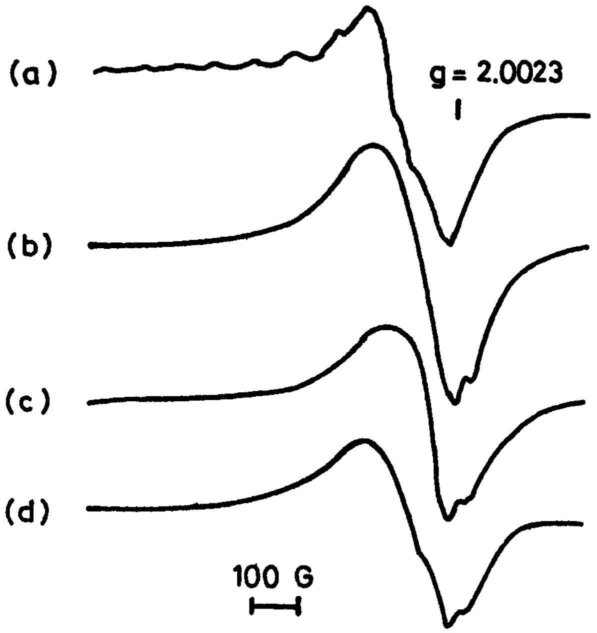

**Figure 6.** EPR spectra of the copper(II) – L-Dopa, – L-adrenaline, – L-DopaP, – DopgP systems at 1:1 metal ion : ligand ratio, pH ca. 7.0, anf 77K.

Formation of a larger ring with DopaP and DopgP can again be explained by steric and electrostatic reasons due to the larger space requirement and higher charge of the phosphonate groups. To understand the dramatic difference in behaviour of the two phosphonate derivatives we can assume, that both substrate (Dopa and DopaP) and inhibitor (DopgP) molecules coordinate to the active centre of tyrosinase via the catecholate binding site with similar strengths, but the differences in charge and steric conditions of the side-chain moiety, which is capable of strong electrostatic binding to the appropriate portion of the enzyme, seems to be the decisive factor in the enzyme-regulatory effect. The phosphonic acid analogue of Dopg most probably acts as a suicide inhibitor. Namely, the enzyme catalyzes its oxidation and the radical intermediate formed exhibits inhibitory action. It is worthwhile to mention that L-Dopa is a widely used prodrug in the treatment of Parkinson disease.[15] In brain it undergoes decarboxylation yielding dopamine, which then acts as neurotransmitter in *substantia nigra*. For therapeutic use L-Dopa must be administered in high daily dose, as a considerable portion of this prodrug is decarboxylated in the peripheral regions, and does not reach the brain. To avoid this extracerebral decarboxylation, L-Dopa is generally used in combination with suitable peripheral L-Dopa decarboxylase inhibitors.[16] It would be interesting to test whether DopaP or some other phosphonic derivative of Dopa can act as a blocker of this enzyme.

Aminopolyphosphonates as multidentate complexing agents with high specificity for various cations are widely used for various purposes. They are applied among others in analytical chemistry, in the paper and textile industries for the removal of trace amounts of metal ions from bleaching baths, and also in medicine for the removal of a metal overload from living organisms.[17-23] A few important representatives of this ligand group can be seen in Figure 7, and their stability constants with several metal ions are listed in Tables II-IV.

The successive protonation constants cannot provide definite information on the protonation sites of the particular species involved in each step. The microscopic constants, obtained mainly via NMR techniques, unambiguously prove that the relative basicity sequence of the donor groups (see above) is overridden by electrostatic factors. A rather complex cyclic configuration between the protons bridging the phosphonate groups and the nitrilo group(s) favourably reduces the repulsive forces between the negatively charged moieties. In some cases, this results in the full protonation of the nitrogens at a lower pH than with the corresponding amino-carboxylates.

These multidentate ligands typically form MA complexes, although molecules of lower denticity (e.g. imino derivatives) can also form bis complexes. Complexes with tri- and tetravalent metal ions may undergo hydrolysis and then olation at higher pH.[5,22,23]

A comparison of the complexing properties of mixed phosphonate and carboxylate ligands (see Table II) indicates that the stability of the complexes increases with the number of phosphonate moieties. In order to demonstrate this, the stabilities of various metal complexes of Nta and its mixed phosphono derivatives are plotted against the number of substituted phosphonate groups in Figure 8. The stability increase is seen to be much lower than would be expected from the increase in basicity of the potentially coordinating groups: the more carboxylate is replaced by phosphonate, the higher the electrostatic and steric hindrance that compensate or overcompensate the former effect.[23] Metal ions such as $Mg^{2+}$, $Ca^{2+}$ and $Fe^{3+}$, which have a higher affinity toward O-donors, display only slight dependences, much less than those which favour both N- and O-coordination. The mutual repulsion between the binegative phosphonate groups tends to lower the coordinating ability and possibly also the denticity of the aminophosphonates with respect to their carboxylate analogues, which results in only a slightly higher, or sometimes even lower, stability of the phosphono complexes of the former metal ions (see Table II).

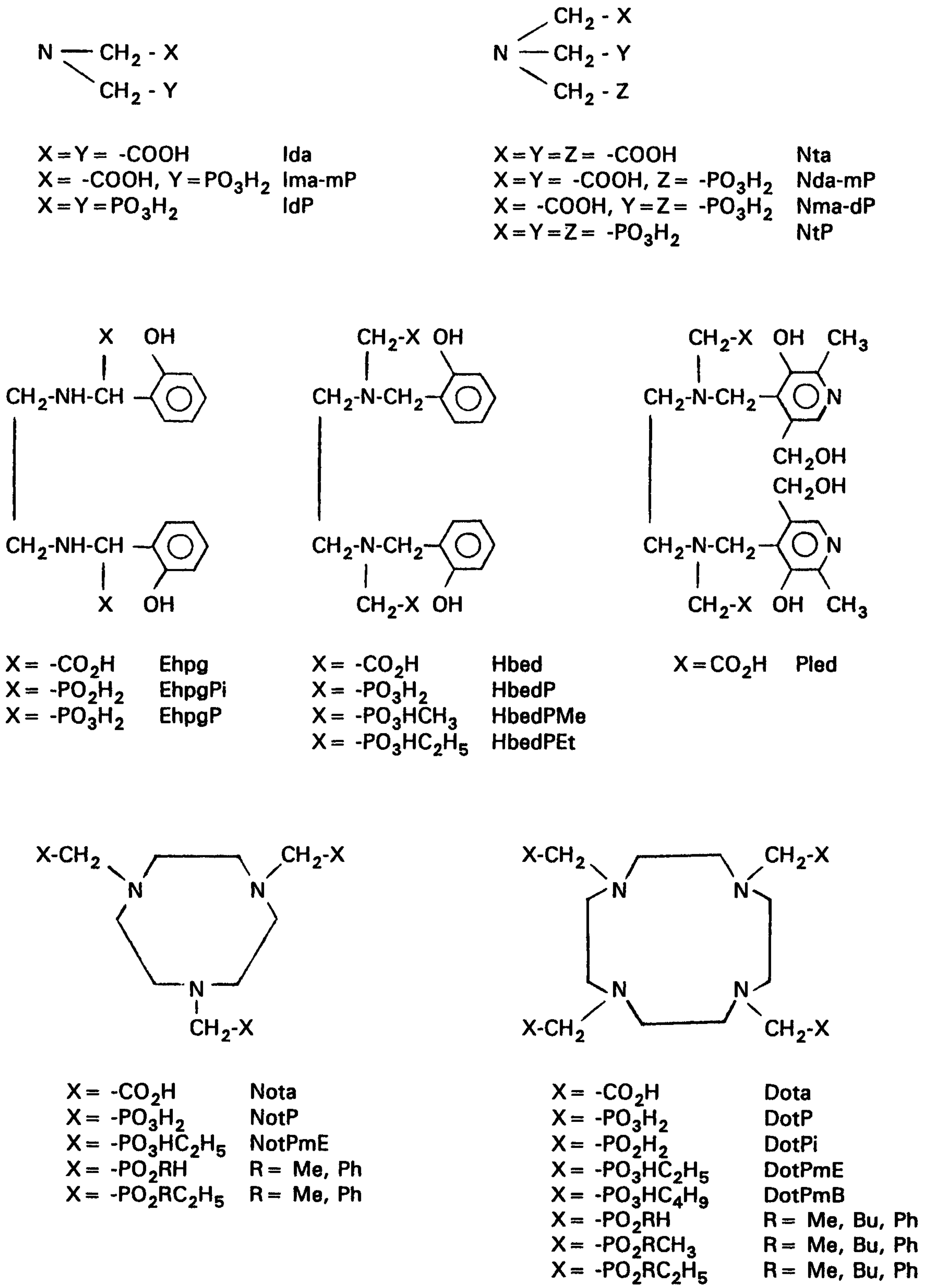

**Figure 7.** Structural formulas of several open-chain and cyclic polyaminopolycarboxylic acids and their phosphonic and phosphinic analogues.

Table II. Log $\beta_{MA}$ values[a] for some mixed carboxylic/phosphonic derivatives of nitrilotriacetic acid (Nta)[b]

| Metal | Nta | Nda-mP | Nma-dP | NtP |
|---|---|---|---|---|
| ΣpK | 14.1 | 20.6 | 26.2 | 32.8 |
| Mg(II) | 5.50 | 6.28 | - | 7.20 |
| Ca(II) | 6.44 | 7.18 | 6.17 | 7.50 |
| Cu(II) | 13.1 | 14.08 | 14.67 | 17.40 |
| Zn(II) | 10.66 | 11.55 | 13.48 | 16.37 |
| Fe(III) | 11.8 | - | 14.65 | 14.60 |

[a] Values are obtained from Ref. 19, 23, 32.
[b] Structural formulas of the ligands are shown in Figure 7. Nda-mP: Nitrilobisacetic-(methylenephosphonic) acid; Nma-dP: Nitriloacetic-bis(methylenephosphonic acid); NtP: nitrilotris(methylenephosphonic acid).

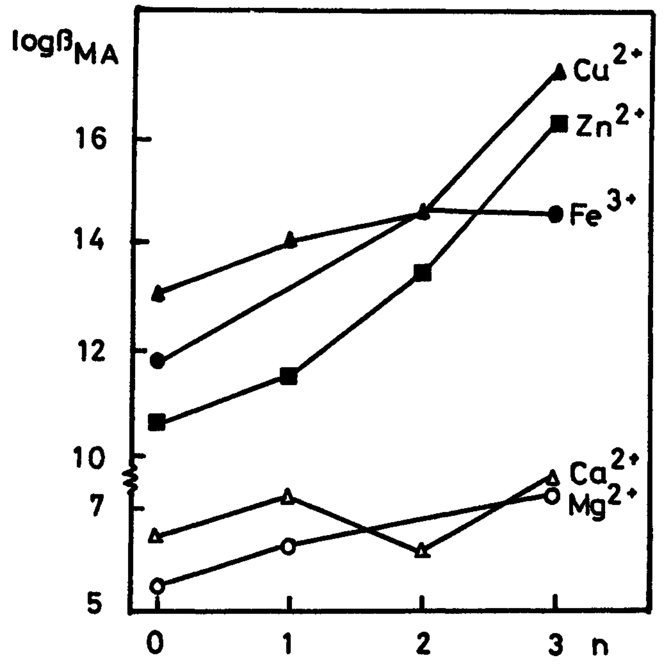

Figure 8. log $\beta_{MA}$ values for metal complexes of mixed phosphonic-carboxylic derivatives of Nta, $N(CH_2PO_3H_2)_n(CH_2COOH)_{3-n}$.

When the coordinating abilities of the bi- and trinegatively charged metal ions are compared, it is seen that the larger positive charge on $Fe^{3+}$ does not reduce significantly the repulsion of the negative phosphonate groups. The reason for this is not clear. It is also seen that the stabilities of the Fe(III) complexes of bis- and trisphosphono derivatives are about the same, which suggests a decrease in denticity of the latter ligand in its complexes.

The high stability of metal complexes of polyaminopolyphosphonates can be utilized in the chelation therapy of metal overload diseases. For instance, the only available treatment of an iron overload resulting from thalassaemia is drug therapy with chelating agents.

Thalassaemias are characterized by various genetically determined defects of globin chain synthesis. The only way to treat the disease-associated anaemia is by regular and frequent blood transfusion. The non-elimination of blood iron and the hyperabsorption of iron from the diet can lead to the accumulation of an enormous amount of iron, which, during long-term treatment, can attain a level of 40-100 g.[24] In order to mobilize the iron the desferrioxamine B (Dfo) of natural origin proved to be very efficient, but the major limitation to its use was its low effectiveness when administered orally and also its rather high cost. In the search for an ideal iron chelator, large number of multidentate ligands have been studied. For example, Ehpg (N,N'-ethylenbis-[2-(o-hydroxyphenyl)methylglycine]) and Hbed (N,N'-di(2-hydroxy-benzyl)-ethylenediamine-N,N'diacetic acid) as well as their phosphono derivatives (some of them are depicted in Figure 7) have been reported[21-23] to form high-stability chelates, due to the high affinity of Fe(III) for the phenolate groups and to the orientation of these groups to form joint chelate systems including the amino and carboxylate/phosphonate functions. The stability constants of the main 1:1 species, formed exclusively at physiological pH in these metal-ligand systems are listed in Table III. Besides the data for Fe(III) and Cu(II) complexes, those for Ga(III) and In(III) are also included in the Table because of their potential applicability as radiopharmaceuticals.

Table III. Log $\beta_{MA}$ values for some phosphonic and phosphinic derivatives of N,N'-ethylenbis-[2-(o-hydroxyphenyl)glycine] (Ehpg)[a]

| Metal | Ehpg | EhpgP | EhpgPi | Hbed | HbedP | HbedPMe | HbedPEt | Pled |
|---|---|---|---|---|---|---|---|---|
| ΣpK | 37.0 | 46.0 | 34.0 | 41.0 | 51.0 | 37.0 | 37.0 | 50.0 |
| Fe(III) | 33.9[b] | 25.0[d] | 31.25[c] | 39.68[e] | prec | 28.21[e] | 28.19[e] | 36.91[b] |
| Ga(III) | 33.6[b] | - | - | 39.57[e] | - | 28.03[e] | 27.98[e] | 36.35[b] |
| In(III) | 33.0[b] | - | - | 39.66[e] | - | 28.12[e] | 28.09[e] | 36.89[b] |
| Cu(II) | 23.94[b] | 18.58[d] | 20.14[c] | 23.69[e] | 24.0[e] | 23.04[e] | 23.01[e] | 19.91[b] |

[a] Structural formulas of the ligands are shown in Figure 7. Ehpg, N,N'-ethylenbis-[2-(o-hydroxyphenyl)glycine]; EhpgP, N,N'-ethylenbis-[2-(o-hydroxyphenyl)methylenephosphonic acid]; EhpgPi, N,N'-ethylenbis-[2-(o-hydroxyphenyl)methylenephosphinic acid]; Hbed, N,N'-di(2-hydroxybenzyl)ethylenediamine-N,N'-diacetic acid; HbedP, N,N'-di(2-hydroxybenzyl)-ethylenediamine-N,N'-(bismethylenephosphonic acid); HbedPMe, N,N'-di(2-hydroxybenzyl)-ethylene-diamine-N,N'-(bismethylenephosphonic acid monomethyl ester); HbedPEt, N,N'-di(2-hydroxybenzyl)ethylenediamine-N,N'-(bismethylenephosphonic acid monoethyl ester); Pled, N,N'-dipyridoxylethylenediamine-N,N'-diacetic acid;
[b] see Ref. 45, [c] see Ref. 28, [d] see Ref. 46, [e] see Ref. 5.

A comparison of the stability constants of the MA complexes shows that phosphonic derivatives form less stable chelates than the analogous aminocarboxylates, even if the overall basicity of the coordinating donors is higher. The complexes, however, are still very stable and formed practically quantitatively at the physiological pH range (pM values are never lower than 25 in solution of 1 mM metal ion and 5 mM ligand at pH 7.4). The reason for the somewhat less favoured formation of phosphono complexes is again the large mutual charge repulsion between the binegative phosphonate groups when more than one such group is coordinated to the same metal ion. In principle, these ligands are typical ambidentate molecules able to form complexes through coordination of the amino and phosphonate/

carboxylate groups or the amino and phenolate groups, or with the simultaneous participation of all six donors. Accordingly, with copper(II) and other bivalent transition metal ions complex formation takes place in two more or less separated steps.[22] In the first, in the acidic pH range, diprotonated complexes $CuH_2A$ are formed containing three joint chelate rings with protonated non-coordinated phenolic-OH groups. With increasing pH, the metal ion begins to compete successfully with the proton for the phenolate groups, and the fully deprotonated complex MA is formed. In CuA, there is probably strong coordination of the metal ion with the amino nitrogens and phenolate oxygens in approximately co-planar positions and weaker coordination with the phosphonate or carboxylate oxygens in the fifth and sixth positions above and below the plane, as clearly indicated by the occurrence of a phenolate O → M charge-transfer band at around 400 nm.[22] It is a general tendency that polyamino-polyphosphono derivatives form protonated complexes more readily, sometimes with similar stability to those of the parent complexes; this strongly suggests that protonation occurs on the non-coordinated phosphonic moieties. This situation is different from that for the aminocarboxylate analogues, where all donor groups are generally coordinated to the metal ion, and thus protonation takes place through proton displacement of the metal ion from the most basic nitrogen site, resulting in a significant decrease in the stability of the protonated complexes. In contrast, with trivalent metal ions all protons from the ligands are displaced in a single step and thus protonated complexes are hardly formed. Complexes with aminopolyphosphinates are generally less stable than both the carboxylate and phosphonate analogues, although partial coordination of the phosphinic groups is supported by both the stability and the spectral data.[1,26-29] Incomplete participation of the donor sites in metal binding, however, as was demostrated for many of the metal – phosphono complexes, may make these ligands more selective for cations with an enhanced capacity for coordination. Because of the high stabilities of the complexes and the fulfilment of the required selectivity at physiological pH, organophosphonates may be promising candidates for the treatment of iron overload anaemias.[5]

The equilibrium, structural and kinetic behaviour of metal complexes of cyclic polyaminopolyphosphonates differs considerably from those of their open-chain counterparts. A few interesting and well-studied representatives of this group are shown in Figure 7. As concerns the acid-base chemistry of these ligands, the cyclic structure modifies the normal basicity sequence (amino being more basic basic than phosphonate) of the donor groups. The protonation equilibria of the aza-N and phosphonate-O donors more or less overlap each other.[30,31] This is fairly well seen in the series of triaza- and tetraazaalicyclicphosphonic acids in which the most basic sites are two ring nitrogens while phosphonate oxygens are less basic and are protonated to different degrees depending on the ring structure. In the tetraaza ligand the protonation of the pendant phosphonate oxygens is more extensive than that in the triaza ligand, before further protonation of the ring nitrogen occurs.[30] The stabilities of some biologically important metal complexes of several triaza- and tetraazaphosphonic derivatives and their carboxylic analogues are shown in Table IV. It can be seen from these data that, besides the basicity of the coordinating donors, as is the case for the open-chain polyaminopolyphosphonates, the size of the ring comprising the metal ion is the main factor governing the metal-binding ability. This is clearly indicated by the fairly high complexation selectivity of the larger $Ca^{2+}$ ion over the smaller $Mg^{2+}$ ion with all tetraaza derivatives and by a reverse selectivity with the triazaalicyclic compounds. Bivalent transition metal ions form considerably more stable complexes due to their greater ability to form metal – nitrogen bonds, than the more ionic alkaline earth metal ions.

Table IV. Log $\beta_{MA}$ values[a] for some aminocarboxylate, -phosphinate and -phosphonate complexes

| Ligand[b] | $\Sigma pK_i$ | Mg(II) | Ca(II) | Sr(II) | Cu(II) | Zn(II) | Cd(II) | Gd(III) |
|---|---|---|---|---|---|---|---|---|
| Nota | 22.0 | 9.7 | 8.9 | - | - | 18.3 | 16.0 | 14.4 |
| NotP | 35.4 | 11.0 | 6.4 | - | 21.3 | 24.9 | 19.7 | - |
| NotPmE | 16.9 | 6.2 | 5.1 | - | - | 15.8 | 13.4 | 10.3 |
| Dota | 30.0 | 11.0 | 15.9 | 12.8 | 19.1 | 18.9 | 19.1 | 24.6 |
| DotP | 43.0 | 7.3 | 10.3 | 9.8 | 22.9 | 24.8 | - | - |
| DotPi | 22.9 | 4.4 | 9.4 | 8.9 | 19.6 | 15.8 | 16.7 | 16.5 |
| DotPmE | 22.1 | 4.6 | 9.1 | 8.9 | - | 14.6 | - | 16.5 |
| DotPmB | 21.4 | 4.3 | 8.5 | 8.1 | - | 14.8 | 18.6 | 12.5 |

[a] Values are obtained from Ref. 47-49.
[b] Structural formulas of the ligands are shown in Figure 7. Nota, 1,4,7-triazacyclononane-triacetic acid; NotP, 1,4,7-triazacyclononanetris(methylenephosphonic acid); NotPmE, 1,4,7-triazacyclononanetris(methylenephosphonic acid monomethyl ester) Dota, 1,4,7,10-tetra-azacyclododecanetetraacetic acid; DotP, 1,4,7,10-tetraazacyclododecanetetrakis-(methylenephosphonic acid); DotPi, 1,4,7,10-tetraazacyclododecanetetrakis(methylene-phosphinic acid); DotPmE, 1,4,7,10-tetraazacyclo-dodecanetetrakis(methylenephosphonic acid monomethyl ester); DotPmB, 1,4,7,10-tetraazacyclododecanetetrakis(methylene-phosphonic acid monobuthyl ester).

A comparison of the stability constants of phosphonic derivatives with those of the tetraaza-ring, cyclen (log $K_{CaA}=3.1$, log $K_{ZnA}=16.21$, log $K_{CdA}=14.3$ and log $K_{CuA}=24.8$),[32] indicates that the side-chain donors contribute substantially to the stability of the earth metal complexes, and only slightly to that of the transition metal complexes. Thus, those metal ions which prefer oxygen coordination are strongly stabilized by the presence of the side-chain O donors, while the stabilities of the transition metal complexes are basically determined by the coordination of the ring nitrogens. All phosphonate derivatives form less stable complexes than do the analogous carboxylates. Although structural data are not available, it is reasonable to assume that $PO_3^{2-}/CO_2^-$ substitution decreases the denticity of coordination, especially to alkaline earth cations, which is manifested in the lower stability of the phosphonates, even if their overall basicity is higher. Macrocyclic aminophosphonates exhibit a much stronger tendency to form oligomeric complexes than the analogous carboxylic compounds.This phenomenon can be easily explained by the steric and electrostatic repulsion between the phosphonate groups. Formation of oligomeric species keeps the bridging binegative phosphonate arms far away from each other minimizing these effects.

Besides the high stability, kinetic inertness makes these macrocyclic complexes particularly suitable for medical applications, for instance in radioimmune therapy[33-38] and magnetic resonance tomography.[7,39,40] In both fields, certain metal ions (such as $In^{3+}$, $Tc^{3+}$, $Cu^{2+}$, $Y^{3+}$, $Au^{3+}$, $Gd^{3+}$) are used in the form of stable complexes of organic ligands either, to bind the metal ions to monoclonal antibody in radioimmun therapy or to decrease their toxicity in NMR tomography. The most popular complex forming agents are open-chain polyaminopolycarboxylates, which are now being increasingly replaced by their macrocyclic analogues, which offer much higher thermodynamic and kinetic stability for the metal complexes. In NMR tomography, $Gd^{3+}$ complexes, having strong paramagnetic properties, are widely used to enhance the contrast of NMR images. Besides the complexes $Gd(Dtpa)^{2-}$

and Gd(Dota)$^-$, which are now approved contrast agents in clinical therapy, intensive research is under way in many laboratories to prepare new derivatives. Phosphonic analogues can be useful in order to improve their applicability, especially in two aspects: to prepare (a) neutral and (b) tissue-specific complexes. The $Gd^{3+}$ complexes of the various tetraaza-phosphonic derivatives, although are less stable than those of the carboxylate analogue DOTA, they are able to bind $Gd^{3+}$ strongly enough to ensure the fast and complete excretion of this toxic metal ion in a reasonably short time after administration. The distribution of some $^{153}$Sm(III)-tetraaza derivatives in the various body tissues 30 minutes after administration is shown in Figure 9.[41] The metal binding ability of Sm(III) is very similar to that of Gd(III), and the use of $^{153}$Sm makes its detection easy.

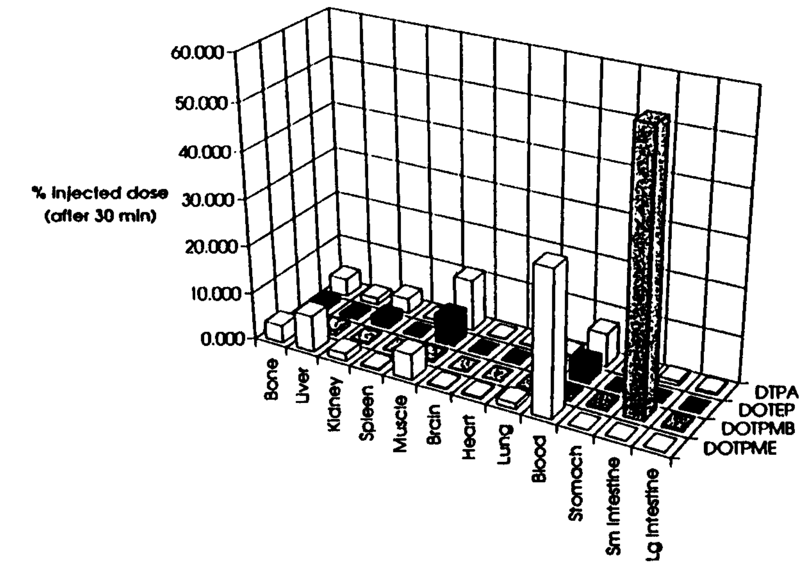

**Figure 9.** Distribution of several $^{153}$Sm(III) complexes in body tissues and fluids of rat 30 minutes after administration.

It can be seen that $^{153}$Sm(Dtpa)$^{2-}$ concentrates mainly in the muscles and the blood, while $^{153}$Sm(DotPmE)$^-$ and $^{153}$Sm(DotPmB)$^-$ are found in the blood and the small intestines, respectively. The corresponding Gd(III) complexes have quite similar biodistribution characteristics in rats. Gd(DotPmB)$^-$ in association with serum albumin shows significantly increased relaxivity, resulting in higher sensitivity than that of any other Gd(III) complex. It is completely excreted from the body through the hepato-biliary system. *In vivo* MRI studies of the Gd(DotPmE)$^-$ and Gd(DotPmB)$^-$ in rabbits exhibited remarkable specificity for liver tissues. Thus, these ligands seem to be most promising candidates for further trials.

Another possible biological importance of these ligands lays in their potential application for measurement of level of certain cations in cells and tissues. Because of the significant selectivity among alkaline earth metal ions (see Table IV), triaza derivatives might be suitable to measure the free $Mg^{2+}$ level, while tetraaza derivatives can be used to measure the free $Ca^{2+}$ concentration in biological systems. Their slow ligand exchange reactions may result in different $^{31}$P NMR signals for the free and metal ion bound species. It was found[42] for example, as shown in Figure 10, that the triazaphosphonic acid ester NOTPmE gave two well-separated $^{31}$P resonances in human red blood cells, corresponding to free and $Mg^{2+}$-bound NotPmE.

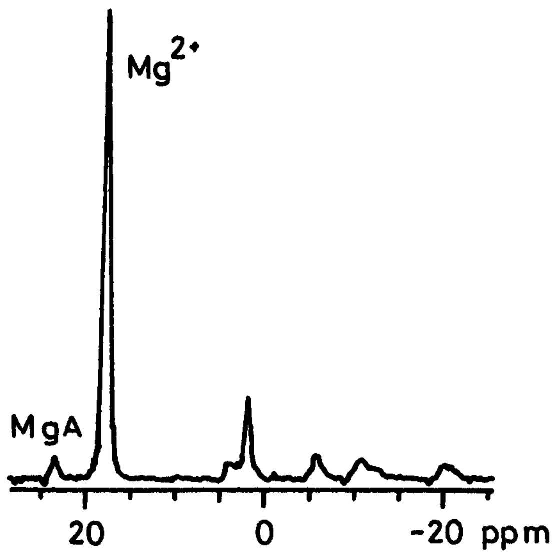

**Figure 10.** $^{31}P$ NMR spectra of NotPmE loaded red blood cells.

Reasonable quantitative concentration values could be calculated by peak integration. Because of the significant selectivity of NotPmE for $Mg^{2+}$ over $Ca^{2+}$ at physiological pH, the $Mg^{2+}$ level could be monitored without interference from $Ca^{2+}$. In principle, tetraaza derivatives, showing a reversed $Ca^{2+}-Mg^{2+}$ selectivity (see Table IV), may be applicable to measure the $Ca^{2+}$ level in biological tissues. Such studies are also now in progress.

**Acknowledgements.** This work was supported in part by grants from the Hungarian Academy of Sciences (Project OTKA 1645/91 and F4026/92).

### References

1 E.N. Rizkalla, *Rev. Inorg. Chem.*, **5**, 223 (1983).
2 P. Kafarski and P. Mastalerz, *Beitr. Wirkst. Forsch.*, **21**, 1 (1984).
3 V.P. Kukhar and V.A. Solodenko, *Usp. Khim.*, **56**, 1504 (1987).
4 P. Kafarski and B. Lejczak, *Phosp. Sulf. Silicon*, **63**, 193 (1991).
5 C.H. Taliaferro and A.E. Martell, *J. Coord. Chem.*, **13**, 249 (1984).
6 R.L. Hayes and K.F. Hubner, *Met. Ions Biol. Syst.*, **16**, 279 (1983).
7 R.B. Lauffer, *Chem. Rev.*, **87**, 901 (1987).
8 T.G. Appleton, J.R. Hall, A.D. Harris, H.A. Kimlin and I.J. McMahon, *Aust. J. Chem.*, **37**,1833 (1984).
9 T.G. Appleton, J.R. Hall and I.J. McMahon, *Inorg. Chem.*, **25**, 720 (1986).
10 E. Matczak-Jon and W. Wojciechowski, *Inorg. Chim. Acta*, **173**, 85 (1990).
11 T. Kiss, *Biocoordination Chemistry*, (ed. K. Burger), Ellis Horwood, Chichester, pp. 104-107.
12 M. Jezowska-Bojczuk, T. Kiss, H. Kozlowski, P. Decock and J. Barycki, *J. Chem. Soc. Dalton*, (submitted).
13 T. Kiss, E. Farkas and H. Kozlowski, *Inorg. Chim. Acta*, **155**, 281 (1989a).

14 J. Balla, T. Kiss, M. Jezowska-Bojczuk, H. Kozlowski and P. Kafarski, *J. Chem. Soc. Dalton,* 1861 (1990).
15 D.B. Calne and M. Sandler, *Nature,* **226,** 21 (1970).
16 B.I. Diamond, K.S. Rajan and R.L. Borison, *Trans. Amer. Neurolog. Assoc.,* **105,** 469 (1981).
17 R.J. Motekaitis, I. Murase and A.E. Martell, *Inorg. Chem.,* **15,** 2303 (1976).
18 E.N. Rizkalla and M.T.M. Zaki, *Talanta,* **26,** 507 (1979); **27,** 423 (1980); **27,** 709 (1980); **27,** 769 (1980).
19 K. Sawada, T. Araki and T. Suzuki, *Inorg. Chem.,* **26,** 1199 (1987).
20 R.P. Carter, R.L. Carroll and R.R. Irani, *Inorg. Chem.,* **6,** 938 (1967).
21 D.T. MacMillan, I. Murase and A.E. Martell, *Inorg. Chem.,* **3,** 469 (1975).
22 C.H. Taliaferro and A.E. Martell, *Inorg. Chem.,* 24, 2408 (1985).
23 R.J. Motekaitis and A.E. Martell, *J. Coord. Chem.,* **14,** 139 (1985).
24 R.R. Crichton, *Inorg. Biochem. of Iron Metabolism,* p183, Ellis Horwood, London, 1991.
25 J. Porter, *Eur. J. Haematol.,* **43,** 271 (1989).
26 R.J. Motekaitis, I. Murase and A.E. Martell, *J. Inorg. Nucl. Chem.,* **33,** 3353 (1971).
27 R.J. Motekaitis, I. Murase and A.E. Martell, *Inorg. Nucl. Chem. Letters,* **7,** 1103 (1971).
28 N.M. Dyatlova, T.Ya. Medved, M.V. Rudomino and M.I. Kabachnik, *Izv. Akad. Nauk SSSR,* **4,** 815 (1970).
29 Yu.F. Belugin, Yu.N. Dubrov, I.N. Marov and N.M. Dyatlova, *Russ. J. Inorg. Chem.,* **17,** 1773 (1972).
30 C.F.G.C. Geraldes, A.D. Sherry and W.P. Cacheris, *Inorg. Chem.,* **28,** 3336 (1989).
31 R. Delgado, L.C. Siegfried and T.A. Kaden, *Helv. Chim. Acta,* **73,** 140 (1990).
32 A.E. Martell and R.M. Smith, *Critical Stability Constants,* Plenum, New York, 1982, Vol V. p183.
33 D. Parker, *Chem. Soc. Rev.,* **19,** 271 (1990).
34 S.V. Deshpande, S.J. DeNardo, D.L. Kukis, M.K. Moi, M.J. McCall, G.L. DeNardo and C.L. Meares, *J. Nucl. Med.,* **31,** 473 (1990).
35 C.J. Broan, K.J. Jankowski, R. Kataky, D. Parker, A.M. Randall and A. Harrison, *J. Chem. Soc. Chem. Commun.,* 1738, 1739 (1990); 204 (1991)
36 E. Cole, D. Parker, G. Ferguson, J.F. Gallagher and B. Kaitner, *J. Chem. Soc. Chem. Commun.,* 1473 (1991).
37 D. Parker, K. Pulukkody, T.J. Norman, A. Harrison, L. Royle and C. Walker, *J. Chem. Soc. Chem. Commun.,* 1441 (1992).
38 C.J. Broan, E. Cole, K.J. Jankowski, D. Parker, K. Pulukkody, B.A. Boyce, N.R.A. Beeley, K. Millar and A.T. Millican, *Synthesis,* 63 (1992).
39 V.M. Runge, D.Y. Gelblum, M.L. Pacetti, F. Carloan and G. Heard, *Radiology,* **177,** 393 (1990); J.-C. Bousquet, S. Saini, D.D. Stark, P.F. Hahn, M. Nigam, J. Wittenberg and J. T. Ferrucci, *Radiology,* **166,** 693 (1988).
40 N. Bansal, M.J. Germann, I. Lázár, C.R. Malloy and A.D. Sherry, *J. Magn. Res. Imag.,* **2,** 385 (1992).
41 C.F.G.C. Geraldes, A.D. Sherry, I. Lázár, A. Miseta, P. Bogner, E. Berényi, B. Sümegi, G.E. Kiefer, K. McMillan, F. Maton and R.N. Muller, *J. Magn. Res. Medicine,* (submitted)
42 R. Ramasamy, I. Lázár, E. Brücher, A.D. Sherry and C.R. Malloy, *Fed. Eur. Biochem. Soc. Letters,* **280,** 121 (1991).
43 T. Kiss, J. Balla, G. Nagy, H. Kozlowski, J. Kowalik, *Inorg. Chim. Acta,* **138,** 25 (1987).
44 T. Kiss, M. Jezowska-Bojczuk, H. Kozlowski, P. Kafarski, K. Antczak, *J. Chem. Soc. Dalton,* 2275 (1991).
45 C.H. Taliaferro, R.J. Motekaitis and A.E. Martell, *Inorg. Chem.,* **23,** 1188 (1984).
46 T.Ya. Medved, M.V. Rudomino, N.M. Dyatlova and M.I. Kabachnik, *Izv. Akad. Nauk. SSSR,* 1211 (1970).
47 M.I. Kabachnik, T.Ya. Medved, Yu.M. Polikarov, B.K. Shcherbakiv, F.I. Belskii, E.I. Matrosov, M.P. Pasechnik, *Izv. Akad. Nauk. SSSR,* 835 (1984).

48 M.I. Kabachnik, T.Ya. Medved, Belskii, S.A. Pisareva, *Izv. Akad. Nauk. SSSR,* 844 (1984).

49 I. Lázár, A.D. Sherry, E. Brücher, R. Király, *Inorg. Chem.,* **30**, 5016 (1991).

**Received: September 3, 1993**

